# Supplementary material for: The manifold actions of signaling peptides on subcellular dynamics of a receptor specify stomatal cell fate
Source: eLife. 2020 Aug 14;9:e58097. doi: 10.7554/eLife.58097 (PMC7470842; doi:10.7554/eLife.58097)
Supplement: Source code 1. [file elife-58097-code1.pdf.zip › Qi_R-scripts_FIN.pdf]

The Manifold Actions of Signaling Peptides on Subcellular Dynamics of a Receptor Specify Stomatal Cell Fate

Xingyun Qi, Akira Yoshinari, Pengfei Bai, Michal Maes, Scott M. Zeng, and Keiko U. Torii

R-scripts generated and used for this study:

Figure 1B:

```
  # Install packages (if needed)
  install.packages("ggplot2")
  install.packages("RColorBrewer")

  # Open libraries needed
  library(ggplot2)
  library(RColorBrewer)
  ERL1marker<-read.csv('ERL1marker.csv')

  #define the first column factors as they are.
  ERL1marker$markers<-as.factor(ERL1marker$markers)

 #Re-assign the order of the boxes
  ERL1marker$markers <- factor(ERL1marker$markers, levels=c("ERL1 vs
Syp43", "Syp43 vs   ERL1", "ERL1 vs Ara7", "Ara7 vs ERL1", "ERL1 vs
Syp22", "Syp22 vs ERL1"), ordered = TRUE)

  #ERL1 marker boxplot---basic settings
  ERL1markerPLOT <- ggplot(ERL1marker, aes(x=markers,y=colocalization,
fill=markers))

  #ERL1 marker boxplot with labels
  PLOT1 <- ERL1markerPLOT + geom_boxplot() + geom_point(size=4) +
theme(axis.text.x = element_text(colour="black", size = 20),
axis.text.y = element_text(colour="black", size = 20),
axis.title=element_text(size=15,face="bold")) + theme(plot.title =
element_text(size = 30, face="bold")) +
    xlab("ERL1 and markers") + ylab("% of colocalization")

  #change colors

  PLOT2 <- PLOT1 + scale_fill_manual(breaks=c("ERL1 vs Syp43", "Syp43
vs ERL1", "ERL1 vs Ara7", "Ara7 vs ERL1", "ERL1 vs Syp22", "Syp22 vs
ERL1"), values = c("red","orange","yellow","green","blue","purple"))

  #show PLOT2
  PLOT2

  # Save as vectormap
  ggsave("ERL1 markers.eps", height=10, width=15)
```

Figure 4C:

```
BFAvolume<-read.csv('BFAvolume_ratio.csv')

#define the first column factors as they are
BFAvolume$treatment<-as.factor(BFAvolume$treatment)

#Re-assign the order of the boxes
BFAvolume$treatment<-factor(BFAvolume$treatment,levels=c("WT-mock",
"tmm-mock", "WT-BFA", "tmm-BFA"), ordered = TRUE)

#ERL1 marker boxplot---basic settings

BFAvolumePLOT <- ggplot(BFAvolume, aes(x=treatment, y=ratioINT,
fill=treatment))

# define comparison between two groups using Kruskal-Wallis,
my_comparisons <- list(c("WT-BFA","tmm-BFA"), c("WT-mock","WT-BFA"),
c("tmm-mock","tmm-BFA"))

#ERL1 marker boxplot with labels
PLOT1 <- BFAvolumePLOT + geom_boxplot() +ylim(0,1)+
geom_jitter(shape=16, size=1, position=position_jitter(0.2)) +
  theme(axis.text.x = element_text(colour="black", size = 10),
axis.text.y = element_text(colour="black", size = 10),
axis.title=element_text(size=20,face="bold")) +
  theme(plot.title = element_text(size = 8, face="bold")) +
  xlab("treatment") + ylab("volume ratio") +
  stat_compare_means(comparison = my_comparisons, method =
"t.test",label = "p.signif") +

#change colors
PLOT2 <- PLOT1 + scale_fill_manual(breaks=c("WT-mock", "tmm-mock",
"WT-BFA", "tmm-BFA"), values =
c("#ffff00","#ff6600","#ffff00","#ff6600"))

#show the PLOT
PLOT2

# Save as vectormap - CHANGE THE FILENAME!!
ggsave("BFAvolumeRatioQuant.eps", height=7, width=5)
```

Figure 4E:

```
WM<-read.csv("ERL1Wm.csv", header=T)

head(WM)
```

```
#define the first column factors as they are
WM$treatment<-as.factor(WM$treatment)

#Re-assign the order of the boxes
WM$treatment<-factor(WM$treatment,levels=c("WTmock", "tmmmock",
"WTWm", "tmmWm"), ordered = TRUE)

#ERL1 marker boxplot---basic settings

WMPLOT <- ggplot(WM, aes(x=treatment,y=Wmbodies, fill=treatment))


#ERL1 marker boxplot with labels
PLOT1W <- WMPLOT + geom_boxplot()+scale_fill_brewer(palette="PuRd") +
geom_jitter(aes(color= treatment), position=position_jitter(0.2),
cex=1.2) + theme(axis.text.x = element_text(colour="black", size
=10),axis.text.y = element_text(colour="black", size =
10),axis.title=element_text(size=10,face="bold")) + theme(plot.title =
element_text(size = 30, face="bold")) +
  xlab("treatment") + ylab("No. of WM bodies")

#show PLOT1
PLOT1W

# Save as vectormap - CHANGE THE FILENAME!!
ggsave("ERL1-WM_KUT.eps", height=8, width=6)


#tmm Wm boxplot with dots with p-values
PLOT4 <- PLOT1 + geom_boxplot() + geom_point(size=1) +
  geom_jitter(shape=16, size=2, position=position_jitter(0.2)) +
  theme(axis.text.x = element_text(colour="black", size = 20),
axis.text.y = element_text(colour="black", size = 20),
axis.title=element_text(size=20,face="bold")) +
  theme(plot.title = element_text(size = 18, face="bold")) +
  xlab("genotype with treatment") + ylab("% of cells with Wm bodies")
+
  stat_compare_means(comparison = my_comparisons, method = "t.test") +


#change colors
PLOT5 <- PLOT4 + scale_fill_manual(breaks=c("WT-mock", "tmm-mock",
"WT-Wm", "tmm-Wm"), values = c("green","red","green","red"))

#show the PLOT
PLOT5

# Save as vectormap - CHANGE THE FILENAME!!
ggsave("tmm Wm with p-values.eps", height=18, width=12)
```

Figure 4H:

```
FRAP<-read.csv("half.csv")

#define the first column factors as they are
FRAP$genotype<-as.factor(FRAP$genotype)

#Re-assign the order of the boxes
FRAP$genotype <- factor(FRAP$genotype, levels=c("WT", "tmm",ordered =
TRUE))

#tmmEPF1 marker boxplot---basic settings,

PLOT1 <- ggplot(FRAP, aes(x=genotype, y=time, fill=genotype))

#tmm EPF1 wiolinplot with dots with labels, y axis limit is from -5 to
20.
PLOT2 <- PLOT1 + geom_boxplot () + geom_point(size=1) +
stat_summary(fun.y = mean,geom = "point",color="cyan") +
theme(axis.text.x = element_text(colour="black", size = 20),
axis.text.y = element_text(colour="black", size = 20),
axis.title=element_text(size=18,face="bold")) + theme(plot.title =
element_text(size = 18, face="bold")) +
  xlab("") + ylab("half time of fluorescence recovery") + ylim (0,100)

#change colors
PLOT3 <- PLOT2 + scale_fill_manual(breaks=c("WT", "tmm"), values =
c("yellow","orange"))

#show the PLOT
PLOT3

#creat a box plot with p-values
PLOT3 + stat_compare_means(method = "t.test")

# Save as vectormap
ggsave("FRAP half time.eps", height=12, width=8)

#try dotplot only
PLOT1+geom_dotplot(binaxis = "y",stackdir="center", stackratio =
0.5,dotsize=0.5)

PLOT2 + scale_fill_hue(l=30, c=100)
```

Figure 4 – figure supplement 1D

```
WMAra<-read.csv("Ara7Wm.csv", header=T)


#define the first column factors as they are
WMAra$treatment<-as.factor(WMAra$treatment)
```

```
#Re-assign the order of the boxes
WMAra$treatment<-factor(WMAra$treatment,levels=c("WTmock", "tmmmock",
"WTWm", "tmmWm"), ordered = TRUE)

#ERL1 marker boxplot---basic settings

WMAraPLOT <- ggplot(WMAra, aes(x=treatment,y=Wmbodies,
fill=treatment))


#ERL1 marker boxplot with labels
PLOT1WA <- WMAraPLOT +
geom_boxplot()+scale_fill_brewer(palette="PuRd") +
geom_jitter(aes(color= treatment), position=position_jitter(0.2),
cex=1.2) + theme(axis.text.x = element_text(colour="black", size
=10),axis.text.y = element_text(colour="black", size =
10),axis.title=element_text(size=10,face="bold")) + theme(plot.title =
element_text(size = 30, face="bold")) +
  xlab("treatment") + ylab("No. of WM bodies")

#show PLOT1
PLOT1WA

# Save as vectormap - CHANGE THE FILENAME!!
ggsave("Ara7WM_KUT.eps", height=8, width=6)
```

Figure 4 – figure supplement 1E

```
endsome<-read.csv("endosomes.csv", header=T)


#define the first column factors as they are
endsome$genotype<-as.factor(endsome$genotype)

#Re-assign the order of the boxes
endsome$genotype<-factor(endsome$genotype,levels=c("ERL1WT",
"ERL1tmm", "Ara7WT", "Ara7tmm"), ordered = TRUE)

#ERL1 marker boxplot---basic settings

endPLOT <- ggplot(endsome, aes(x=genotype,y=endosomes, fill=genotype))


#ERL1 marker boxplot with labels
PLOTendosome <-endPLOT +
geom_boxplot()+scale_fill_brewer(palette="PuRd") +
geom_jitter(aes(color= genotype), position=position_jitter(0.2),
cex=1.2) + theme(axis.text.x = element_text(colour="black", size
=10),axis.text.y = element_text(colour="black", size =
```

```
10),axis.title=element_text(size=10,face="bold")) + theme(plot.title =
element_text(size = 30, face="bold")) +
  xlab("genotype") + ylab("No. endosomes")

#show PLOT1
PLOTendosome

# Save as vectormap - CHANGE THE FILENAME!!
ggsave("PLOTendosome_KUT.eps", height=8, width=12)
```

Figure 4 – figure supplement 2

```
CHXBFA<-read.csv("dKCHXBFA.csv", header=T)

#define the first column factors as they are

CHXBFA$treatment<-as.factor(CHXBFA$treatment)

#Re-assign the order of the boxes

CHXBFA$treatment<-factor(CHXBFA$treatment,levels=c("WTCHXmock",
"tmmCHXmock", "WTCHXBFA", "tmmCHXBFA", "JM259CHXmock", "JM259CHXBFA"),
ordered = TRUE)

#ERL1 marker boxplot---basic settings

CHXPLOT <- ggplot(CHXBFA, aes(x=treatment,y=bodies, fill=treatment))

#ERL1 marker boxplot with labels

PLOT1 <- CHXPLOT + geom_boxplot()+scale_fill_brewer(palette="PuRd") +
geom_jitter(aes(color= treatment), position=position_jitter(0.2),
cex=1.2) + theme(axis.text.x = element_text(colour="black", size
=10),axis.text.y = element_text(colour="black", size =
10),axis.title=element_text(size=10,face="bold")) + theme(plot.title =
element_text(size = 30, face="bold")) +

  xlab("treatment") + ylab("No. of BFA bodies") +stat_summary(fun
=mean, geom = "point", shape = 18, size = 5, color = "black")

#show PLOT1

PLOT1

ggsave("CHXall_KUT.eps", height=8, width=10)
```

Figure 8E:

```
JM259<-read.csv("JM259.csv")

summary(JM259)


#define the first column factors as they are.

JM259$treatment<-as.factor(JM259$treatment)

#Re-assign the order of the boxes

JM259$treatment <- factor(JM259$treatment, levels=c("mockBFA", "BFA",
"mockWm", "Wm",ordered = TRUE))


#ERL1 marker boxplot---basic settings

JM259PLOT <- ggplot(JM259, aes(x=treatment, y=bodies, fill=treatment))
+ geom_violin (trim=TRUE)

# define comparison between two groups using anova,

my_comparisons <- list(c("mockBFA","BFA"), c("mockWm","Wm"))

compare_means(bodies ~ treatment, data = JM259, method = "anova")

# Violin with dots (stat by default is Kruskal.test, change method to
anova by adding <method="anova">, <label="p.signif"> changes the
absolute p-value to stars)

JM259PLOT2<-JM259PLOT + stat_summary (fun.y=mean, geom="point",
shape=23, size=2) +

  geom_boxplot(width=0.1) + geom_jitter(shape=16, size=2,
position=position_jitter(0.2)) +

  theme(axis.text.x = element_text(colour="black",
size=20),axis.text.y = element_text(colour="black",
size=10),axis.title=element_text(size=20,face="bold")) + xlab("") +
ylab("No of endosome bodies per cell") + ylim(-1,5) +

  stat_compare_means(comparison = my_comparisons, method =
"t.test",label = "p.signif") + stat_compare_means(method = "anova",
label.y = 5)

#Violin without dots

JM259PLOT3<-JM259PLOT + stat_summary (fun.y=mean, geom="point",
shape=23, size=2) +

  geom_boxplot(width=0.1) +

  theme(axis.text.x = element_text(colour="black",
size=20),axis.text.y = element_text(colour="black",
```

```
size=10),axis.title=element_text(size=20,face="bold")) + xlab("") +
ylab("No of endosome bodies per cell")+ ylim(-1,5) +

  stat_compare_means(comparisons = my_comparisons,method =
"t.test",label = "p.signif") + stat_compare_means(method = "anova",
label.y = 5)

#change colors

JM259PLOT4 <- JM259PLOT2 + scale_fill_manual(breaks=c("mockBFA",
"BFA", "mockWm", "Wm"), values = c("red","yellow","green","blue"))

JM259PLOT5 <- JM259PLOT3 + scale_fill_manual(breaks=c("mockBFA",
"BFA", "mockWm", "Wm"), values = c("red","yellow","green","blue"))

JM259PLOT4

JM259PLOT5


# Save as vectormap

ggsave("JM259 with dots and asterisk.eps", height=10, width=12)

ggsave("JM259 without dots and asterisk.eps", height=10, width=12)
```

Figures 5E, F, 6E, F:

```
# Install packages (if needed)

install.packages("ggplot2")

install.packages("RColorBrewer")

install.packages("Hmisc")

install.packages("ggpubr")


# Open libraries needed

library(ggplot2)

library(RColorBrewer)


#load ggpubr

library(ggpubr)


EPFL6<-read.csv("tmm mEPFL6.csv")
```

```
#define the first column factors as they are

EPFL6$mEPFL6<-as.factor(EPFL6$mEPFL6)


#Re-assign the order of the boxes

EPFL6$mEPFL6 <- factor(EPFL6$mEPFL6, levels=c("mock", "1uM", "2.5uM",
"5uM",ordered = TRUE))


#tmmEPF1 marker boxplot---basic settings,

PLOT1 <- ggplot(EPFL6, aes(x=mEPFL6, y=endosomes, fill=mEPFL6))


#tmm EPF1 wiolinplot with dots with asterisks, y axis limit is from -5
to 20.

PLOT2 <- PLOT1 + geom_violin(trim = TRUE) + geom_boxplot (width=0.1) +
geom_jitter(shape=16, size=2, position=position_jitter(0.2)) +

  stat_summary(fun.data = "mean_sdl",fun.args = list(mult=1),geom =
"pointrange",color="yellow") +

  theme(axis.text.x = element_text(colour="black", size = 12),
axis.text.y = element_text(colour="black", size = 10),
axis.title=element_text(size=18,face="bold")) + theme(plot.title =
element_text(size = 18, face="bold")) +

  xlab("mEPFL6 concentration") + ylab("NO. of endosomes per cell") +
ylim (-1.5,17.5) +

  stat_compare_means(method = "t.test",label = "p.signif",ref.group =
"mock") + stat_compare_means(method = "anova", label.y = 17)


#change colors

PLOT3 <- PLOT2 + scale_fill_manual(breaks=c("mock", "1uM", "2.5uM",
"5uM"), values = c("pink","orange","red","purple"))


#show the PLOT

PLOT3


#save to eps file

ggsave("tmm mEPFL6 with asterisks.eps", height = 10, width = 12)
```

```
#tmm EPF1 wiolinplot with dots with asterisks, y axis limit is from -5 to 20.

PLOT2 <- PLOT1 + geom_violin(trim = TRUE) + geom_boxplot (width=0.1) + geom_jitter(shape=16, size=2, position=position_jitter(0.2)) +

  stat_summary(fun.data = "mean_sdl",fun.args = list(mult=1),geom = "pointrange",color="yellow") +

  theme(axis.text.x = element_text(colour="black", size = 12), axis.text.y = element_text(colour="black", size = 10), axis.title=element_text(size=18,face="bold")) + theme(plot.title = element_text(size = 18, face="bold")) +

  xlab("mEPFL6 concentration") + ylab("NO. of endosomes per cell") + ylim (-1.5,17.5) +

  stat_compare_means(method = "t.test",ref.group = "mock") + stat_compare_means(method = "anova", label.y = 17)

#change colors

PLOT3 <- PLOT2 + scale_fill_manual(breaks=c("mock", "1uM", "2.5uM", "5uM"), values = c("pink","orange","red","purple"))


#show the PLOT

PLOT3

#save to eps file

ggsave("tmm mEPFL6 with p-values.eps", height = 10, width = 12)
```

Figure 6 -figure supplement 1B

```
epf1BFA<-read.csv('epf1BFA.csv')

head(epf1BFA)

#define the first column factors as they are
epf1BFA$treatment<-as.factor(epf1BFA$treatment)

#Re-assign the order of the boxes
epf1BFA$treatment<-factor(epf1BFA$treatment,levels=c("WTmock", "epf1mock", "WTBFA", "epf1BFA"), ordered = TRUE)

#ERL1 marker boxplot---basic settings

epf1BFAPLOT <- ggplot(epf1BFA, aes(x=treatment,y=BFAbodies, fill=treatment))
```

```
#ERL1 marker boxplot with labels
PLOT1 <- epf1BFAPLOT + geom_boxplot() + geom_point(size=2) +
theme(axis.text.x = element_text(colour="black", size =10),axis.text.y
= element_text(colour="black", size =
10),axis.title=element_text(size=10,face="bold")) + theme(plot.title =
element_text(size = 30, face="bold")) +
  xlab("treatment") + ylab("No. of BFA bodies")

#show PLOT1
PLOT1

# Save as vectormap - CHANGE THE FILENAME!!
ggsave("epf1BFA.eps", height=10, width=15)
```

Figure 5 -figure supplement 1C

```
epf1Wm<-read.csv("epf1Wm.csv", header=T)

head(epf1Wm)

#define the first column factors as they are
epf1Wm$treatment<-as.factor(epf1Wm$treatment)

#Re-assign the order of the boxes
epf1Wm$treatment<-factor(epf1Wm$treatment,levels=c("WTmock",
"epf1mock", "WTWm", "epf1Wm"), ordered = TRUE)

#ERL1 marker boxplot---basic settings

epf1WmPLOT <- ggplot(epf1Wm, aes(x=treatment,y=Wmbodies,
fill=treatment))


#ERL1 marker boxplot with labels
PLOT1 <- epf1WmPLOT + geom_boxplot()+scale_fill_brewer('alette="PuRd")
+ geom_jitter(aes(color= Genotype), position=position_jitter(0.2),
cex=1.2) + theme(axis.text.x = element_text(colour="black", size
=10),axis.text.y = element_text(colour="black", size =
10),axis.title=element_text(size=10,face="bold")) + theme(plot.title =
element_text(size = 30, face="bold")) +
  xlab("treatment") + ylab("No. of Wm bodies")

#show PLOT1
PLOT1

# Save as vectormap - CHANGE THE FILENAME!!
ggsave("epf1Wm.eps", height=10, width=15)
```

Welch’s Two Sample T-tests (Unpaired) -basic scripts

```
myttest <- read.csv("BFA-vol-ttest.csv", header =T)

t.test(myttest$WT, myttest$WTBFA, paired=F)

t.test(myttest$WT, myttest$tmm, paired=F)

t.test(myttest$WT, myttest$tmmBFA, paired=F)
```

Two-Way Anova and Tukey's HSD test -basic scripts

```
install.packages("stats")

#Two-way ANOVA

Tway <-read.csv("Fig6-figsup1C-2way.csv", header=T)

anova2 <-lm(bodies~genotype*treatment, data=Tway)
anova2

plot(density(anova2$residuals))

qqnorm(anova2$residuals)

plot(anova2$residuals~anova2$fitted.values)

anova(anova2)

Tukey<-aov(bodies~genotype*treatment, data=Tway)

TukeyHSD(Tukey, which="treatment")
TukeyHSD(Tukey, which="genotype")
```
