# Supplementary material for: The manifold actions of signaling peptides on subcellular dynamics of a receptor specify stomatal cell fate
Source: eLife. 2020 Aug 14;9:e58097. doi: 10.7554/eLife.58097 (PMC7470842; doi:10.7554/eLife.58097)
Supplement: Supplementary file 1. [file elife-58097-supp1.docx]

**The Manifold Actions of Signaling Peptides on Subcellular Dynamics of a Receptor Specify Stomatal Cell Fate**

Xingyun Qi, Akira Yoshinari, Pengfei Bai, Michal Maes, Scott M. Zeng, and Keiko U. Torii

**Supplementary Table 1: List of DNA primers and their sequence used in the study**

| *er105*  (Shpak et al.,2015) | WT band | AAGAAGTCATTCAAAGATGTGA & AGAATTTTCAGGTTTGGAATCTGT |
| --- | --- | --- |
|  | Mutant band | AAGAAGTCATTCAAAGATGTGA & AGCTGACTATACCCGATACTGA |
| *erl1-2*  (Shpak et al.,2015) | WT band | GAGCTTGGACATATAATCAATC & CCGGAGAGATTGTTGAAGGA |
|  | Mutant band | GTCACGTCTCAGCTATTTGTAAGCTTGTT & CATTTTATAATAACGCTGCGGACATCTAC |
| *erl2-1*  (Shpak et al.,2015) | WT band | GCCTATTCCACCAATACTTG & ACAAATCTGAGAGAGTTAATGCAAAGCAG |
|  | Mutant band | ACAAATCTGAGAGAGTTAATGCAAAGCAG & CATTTTATAATAACGCTGCGGACATCTAC |
| *tmm-KO*  (Hara et al.,2007) | WT band | CTCAAACACCTCAAAGCCTT &  GAACCGAATGCATCATCCAAGTCACT |
|  | Mutant band | ATTTTGCCGATTTCGGAAC & GAACCGAATGCATCATCCAAGTCACT |
| *epf1*  (Hara et al.,2007) | WT band | GTTAAGCCGTTGACTTTGG &  CGGAGCTCAGGGACAGGGTGGACTTAT |
|  | Mutant band | ATTTTGCCGATTTCGGAAC &  CGGAGCTCAGGGACAGGGTGGACTTAT |
