# Supplementary material for: The manifold actions of signaling peptides on subcellular dynamics of a receptor specify stomatal cell fate
Source: eLife. 2020 Aug 14;9:e58097. doi: 10.7554/eLife.58097 (PMC7470842; doi:10.7554/eLife.58097)
Supplement: Transparent reporting form [file elife-58097-transrepform.docx]

***eLife’s* transparent reporting form**

We encourage authors to provide detailed information *within their submission* to facilitate the interpretation and replication of experiments. Authors can upload supporting documentation to indicate the use of appropriate reporting guidelines for health-related research (see [EQUATOR Network](http://www.equator-network.org/%20)), life science research (see the [BioSharing Information Resource](https://biosharing.org/)), or the [ARRIVE guidelines](http://www.plosbiology.org/article/info:doi/10.1371/journal.pbio.1000412) for reporting work involving animal research. Where applicable, authors should refer to any relevant reporting standards documents in this form.

If you have any questions, please consult our Journal Policies and/or contact us: [editorial@elifesciences.org](mailto:editorial@elifesciences.org).

**Sample-size estimation**

- You should state whether an appropriate sample size was computed when the study was being designed
- You should state the statistical method of sample size computation and any required assumptions
- If no explicit power analysis was used, you should describe how you decided what sample (replicate) size (number) to use

Sample size was determined based on similar studies in this field.

Figure 1: The numbers of cells analyzed for Fig. 1C were stated in the figure legend. R ggplot2 was used to generate boxplot graph. Individual data points with exact raw values are overlaid as dot plots.

Figure 1 - figure s1- Provides a justification for Wm bodies vs regular endosomes by the factor of diameter. Welch's two sample t-test hasbeen performed.

Figure 2: Experiments were performed with three independent seedlings.

Figure 3: Experiments in A, B were performed with a single seedling, and C were performed in three or four independent seedlings in triplicates.

Figure 3 – figure s1: All experiments were performed with three or four independent seedlings in triplicates.

Figure 4: The numbers of cells analyzed for Fig. 4D, 4F and 4H were stated in the figure legend. R ggplot2 was used to generate boxplot graph. Welch’s Two Sample T-test was performed for pairwise comparisons. p value was provided. For Fig. 4G, the R-based FrapBot software was used to fit the FRAP recovery curves to a single-parameter exponential model for the half time determination. Individual data points with exact raw values are overlaid as dot plots.

Figure 4- figure s1: The numbers of cells analyzed for figure. 4 -- figure s1 were stated in the figure legend. R ggplot2 was used to generate boxplot graph. Individual data points with exact raw values are overlaid as dot plots with jitter value of 0.2. Welch’s Two Sample T-test was performed for pairwise comparisons. Two-way ANOVA has been performed to test the genotype: treatment effects. p value was provided.

Figure 4- figure s2: The plots of the fluorescence intensity along the line drawn in the corresponding images in Fig. 4 – figure s1A were generated in Excel. The numbers of cells analyzed for figure. 4 – figure s2D, s1E were stated in the figure legend. R ggplot2 was used to generate boxplot graph. Individual data points with exact raw values are overlaid as dot plots with jitter value of 0.2. Welch’s Two Sample T-test was performed for pairwise comparisons. Two-way ANOVA has been performed to test the genotype: treatment effects. p value was provided.

Figure 4- figure s3: The plots of the fluorescence intensity of FRAP shown in Fig. 4 – figure s3 were generated in Excel.

Figure 5- figure s1: The numbers of cells analyzed for figure6. S1B, s1C were stated in the figure legend. R ggplot2 was used to generate boxplot graph. Individual data points with exact raw values are overlaid as dot plots with jitter value of 0.2. Welch’s Two Sample T-test was performed for pairwise comparisons. Two-way ANOVA has been performed to test the genotype: treatment effects. p value was provided.

Figure 6: The numbers of cells analyzed for Fig. 7E and 7F were stated in the figure legend. R ggplot2 was used to generate violin plot graph in Fig 7E and 7F. Individual data points with exact raw values are overlaid as dot plots with jitter value of 0.2. Welch’s Two Sample T-test was performed for pairwise comparisons. p value was provided.

Figure 6 – figure s1: For Fig7. s1B and s1C, the numbers of cells analyzed were stated in the figure legend, the dose response curves were fitted to the generalized log logistic distribution using the R-package 'drc'.

Figure 7. The plots of the fluorescence intensity along the line drawn in the corresponding images in Fig. 8B and 8C were generated in Excel.

Figure 7 – figure s2: 3 seedlings were analyzed for each treatment in Figure. 8 – figure s2. Experiments were repeated twice independently.

Figure 7 – figure s3: 3 seedlings were analyzed for each treatment in Figure. 7 – figure s3A, s3B. Experiments were repeated four times independently.

(Continued) Sample size was determined based on similar studies in this field.

Figure 8: The numbers of cells analyzed for Fig. 8-figS1 were stated in the figure legend. R ggplot2 was used to generate violin plot graph. Individual data points with exact raw values are overlaid as dot plots with jitter value of 0.2. Welch’s Two Sample T-test was performed for pairwise comparisons. p value was provided.

Please outline where this information can be found within the submission (e.g., sections or figure legends), or explain why this information doesn’t apply to your submission:

**Replicates**

- You should report how often each experiment was performed
- You should include a definition of biological versus technical replication
- The data obtained should be provided and sufficient information should be provided to indicate the number of independent biological and/or technical replicates
- If you encountered any outliers, you should describe how these were handled
- Criteria for exclusion/inclusion of data should be clearly stated
- High-throughput sequence data should be uploaded before submission, with a private link for reviewers provided (these are available from both GEO and ArrayExpress)

Please outline where this information can be found within the submission (e.g., sections or figure legends), or explain why this information doesn’t apply to your submission:

To ensure robust reproducibility: the pharmacological data presented in this manuscript were repeated three times. FRAP experiment was repeated three times. All confocal images presented were imaged at least six times for a single data point. For transgenic lines: at least three individual lines for each transgenic plant were analyzed before carrying out further experiments with the lines.

**Statistical reporting**

- Statistical analysis methods should be described and justified
- Raw data should be presented in figures whenever informative to do so (typically when N per group is less than 10)
- For each experiment, you should identify the statistical tests used, exact values of N, definitions of center, methods of multiple test correction, and dispersion and precision measures (e.g., mean, median, SD, SEM, confidence intervals; and, for the major substantive results, a measure of effect size (e.g., Pearson's r, Cohen's d)
- Report exact p-values wherever possible alongside the summary statistics and 95% confidence intervals. These should be reported for all key questions and not only when the p-value is less than 0.05.

Please outline where this information can be found within the submission (e.g., sections or figure legends), or explain why this information doesn’t apply to your submission:

All statistical methods and individual number of samples analyzed are indicated in Figure legends. For pairwise comparison, Welch's two-sample t test were used throughout. For genotype vs treatment experiments, two-way ANOVA was in addition performed. Tukey's HSD post hoc test was subsequently performed. For box plots, violin plots, and dose-response curve, the exact individual data points are plotted as dots (with a jitter for violin plots: geometric gitter =0.2). Detailed response to individual figures will largely repetitive from our response to Sample size estimation (All points are addressed above. See above for specific statistical analyses done for each figure panels).

(For large datasets, or papers with a very large number of statistical tests, you may upload a single table file with tests, Ns, etc., with reference to sections in the manuscript.)

**Group allocation**

- Indicate how samples were allocated into experimental groups (in the case of clinical studies, please specify allocation to treatment method); if randomization was used, please also state if restricted randomization was applied
- Indicate if masking was used during group allocation, data collection and/or data analysis

Please outline where this information can be found within the submission (e.g., sections or figure legends), or explain why this information doesn’t apply to your submission:

NA

**Additional data files (“source data”)**

- We encourage you to upload relevant additional data files, such as numerical data that are represented as a graph in a figure, or as a summary table
- Where provided, these should be in the most useful format, and they can be uploaded as “Source data” files linked to a main figure or table
- Include model definition files including the full list of parameters used
- Include code used for data analysis (e.g., R, MatLab)
- Avoid stating that data files are “available upon request”

Please indicate the figures or tables for which source data files have been provided:

R Scripts for Figure1C; Figure 4D, 4F, 4H; Figure 4-s1, Figure 4-s2D, 4-s1E, Figure 5E, 5F, Figure 5-s1B, 5-s1C, Figure 6E, 6F, Figure 6-s1B, 6-s1C, Figure 8-figure supplement1 are provided as a source file (Qi_R-scripts_Final.pdf).
